# Small bowel haemorrhage associated with partial midgut malrotation in a middle-aged man

**DOI:** 10.1186/1749-7922-4-1

**Published:** 2009-01-13

**Authors:** Ajay Belgaumkar, Dheeraj Karamchandani, Praveen Peddu, Klaus-Martin Schulte

**Affiliations:** 1 Department of General Surgery, King's College Hospital, Denmark Hill, London, UK; 2 Department of Radiology, King's College Hospital, Denmark Hill, London, UK; 3 King's College London, University of London, UK

## Abstract

We describe a case of life-threatening small bowel haemorrhage in a 56 year old man, who was found to have partial midgut malrotation at laparotomy. An association between congenital malrotation and gastrointestinal haemorrhage has not previously been reported in this age group. We discuss the association between gut malrotation and small intestinal pathology and describe the principles of management in these patients.

## Background

Gastrointestinal haemorrhage is a common acute presentation to emergency hospital services. The commonest causes of bleeding from the upper gastrointestinal tract are secondary to peptic ulceration or complications of portal hypertension [1]. Colonic sources of bleeding include diverticular disease, neoplasia and angiodysplasia[2]. Initial treatment of these patients involves cardiovascular resuscitation, stabilisation of coagulopathy, followed by endoscopic examination of the upper gastrointestinal tract up to the second part of the duodenum and colonoscopy. Significant haemorrhage from the small intestine is relatively uncommon and may create difficulties in diagnosis and treatment[3].

We present a case of small intestinal haemorrhage that was managed by emergency laparotomy, discuss the likely aetiology of the haemorrhage and the principles of management in these groups of patients.

## Case Presentation

A 56 year old man presented to the Emergency Department after passing bright red blood mixed with dark clots per rectum. He had vague, crampy abdominal pains for the previous two days. Past medical history included hypertension, type 2 diabetes and ischaemic heart disease. One year previously, he was admitted to hospital with vague, intermittent central abdominal pain, which resolved following observation for 5 days.

On admission, he was tachycardic and hypotensive, with no abdominal tenderness or palpable masses. Rectal examination revealed bright red blood and clots on the glove. Admission haemoglobin was 8 g/dl. Serum ferritin was low at 19 μg/L. He was resuscitated and stabilised with intravenous fluids. Computed tomography (CT) scan demonstrated uncomplicated sigmoid diverticular disease and no other pathology to explain his symptoms. He underwent urgent upper gastrointestinal endoscopy, which was normal to the second part of the duodenum, with no signs of haemorrhage. Subsequent colonoscopy showed a colon full of fresh blood and clots up to the caecum, with no obvious bleeding source. Intubation of the small bowel and examination of the terminal ileum showed fresh blood filling the lumen, with a likely bleeding point in the proximal small bowel beyond the reach of the endoscope. At this stage, the patient became haemodynamically unstable and a decision was made to take the patient for an urgent exploratory laparotomy.

**Figure 1:**
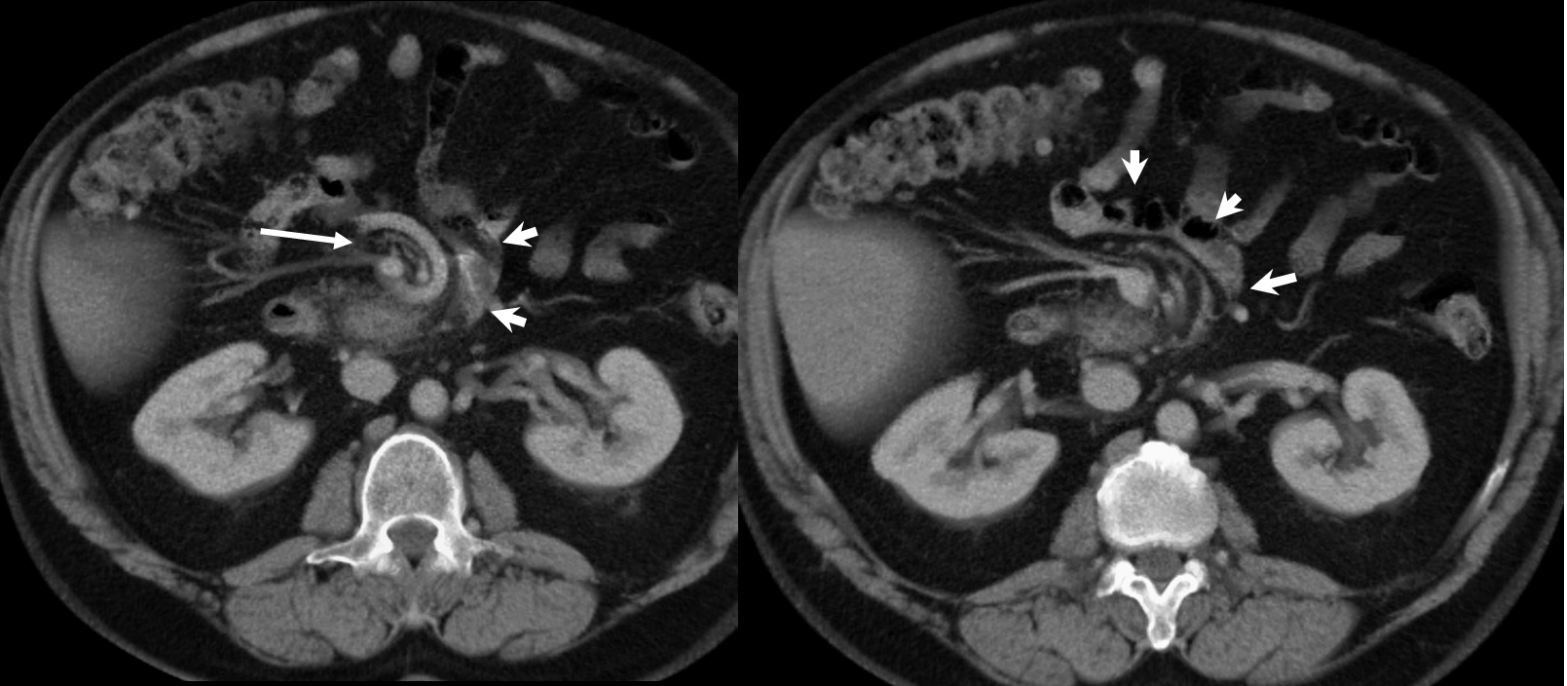
**Contrast enhanced CT axial images at the level of L2 demonstrating abnormal rotation of the proximal jejunum (short arrows)**. Note the swirling of the superior mesenteric vein (long arrow).

**Figure 2:**
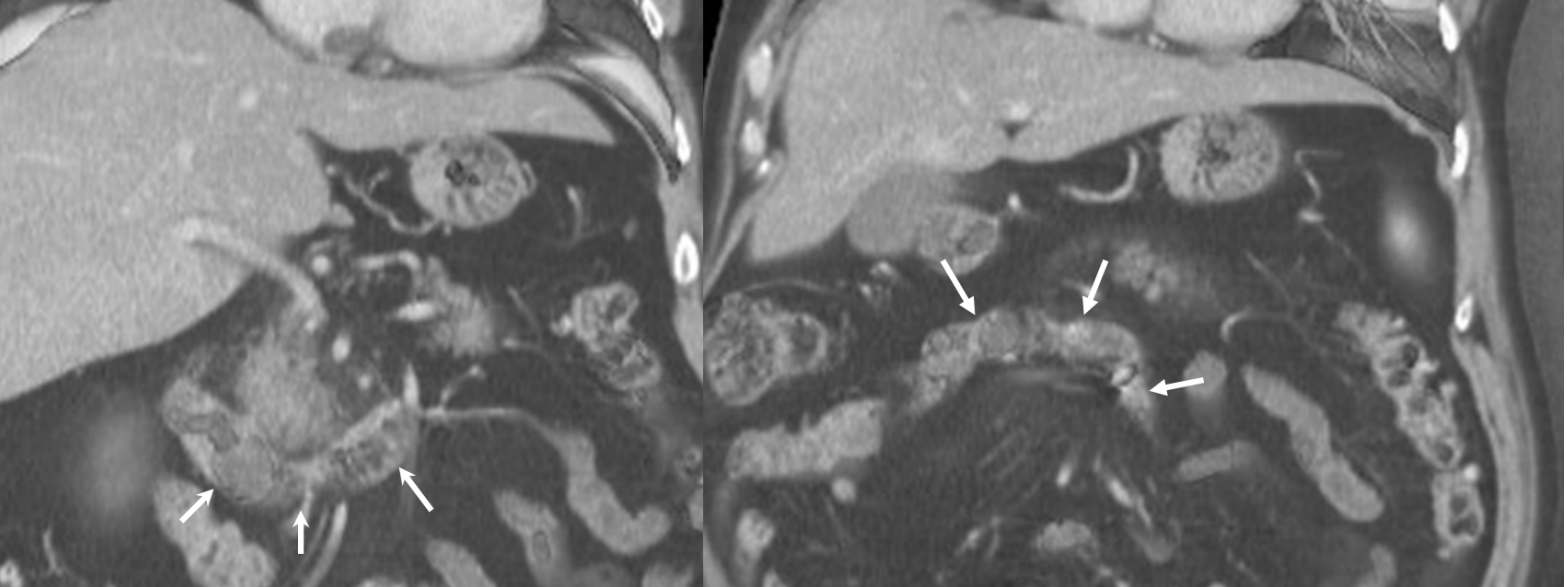
**CT, coronal reformatted images demonstrating abnormal rotation of the proximal jejununum, with proximal segment extending horizontally across the midline to the right side of the abdomen (arrows)**.

At laparotomy, blood was seen to fill the entire large intestine. The small bowel was filled with blood from the terminal ileum up to the proximal jejunum. The first 100 cm jejunum, after the ligament of Trietz, was fixed to the retroperitoneum with the rest of the proximal jejunum lying to the right of the midline (Figures 1 &2). There were no palpable masses or visible inflammatory pathology. The bleeding source was presumed to be in the proximal jejunum. The blood in the small bowel was emptied manually and a series of soft bowel clamps were applied to observe and confirm the site of the bleed. Blood was seen to fill the proximal jejunum, in the segment which was abnormally fixed in the retroperitoneum. The malrotated segment of jejunum was mobilised from the retroperitoneum. A segmental small bowel resection (75 cm) was performed, centred on the presumed point of haemorrhage. A primary side-to-side jejeno-jejeunal anastomosis was fashioned. The small bowel was examined again, with no further haemorrhage noted.

**Figure 3:**
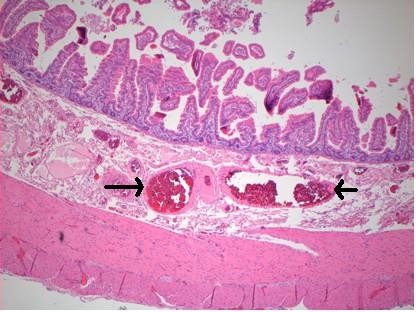
**Histological examination demonstrates dilated blood vessels within the submucosa (arrows)**.

Six units of blood were transfused during the operation. The patient was managed on the high dependency unit for 48 hours and was transferred to the surgical ward. His recovery was complicated by an infection of his central venous catheter site and Clostridium difficile-associated diarrhoea. He was discharged 14 days following surgery, with no evidence of further gastrointestinal bleeding or cardiovascular instability. Histological examination of the resected small bowel demonstrated focal dilatation of vessels within the mucosa, submucosa and muscularis propria layers, with areas of erosion, in keeping with the likely source of haemorrhage (Figure 3). There was no evidence of thrombosis, vasculitis or neoplasia. The patient remained well at three month follow-up with no further drop in haemoglobin or signs of gastrointestinal bleeding.

## Discussion

An association between congenital malrotation of the midgut and life-threatening gastrointestinal bleeding has not been previously reported in patients over 50 years of age.

In patients aged above 50, angiodysplasia occurs with greater frequency and may present as intermittent gastrointestinal bleeding, most commonly with iron deficiency anaemia with normal upper and lower gastrointestinal endoscopy[4]. Haemodynamically stable patients are amenable to further investigation, which may include capsule endoscopy, CT angiography and percutaneous selective mesenteric angiography[3]. These investigations are time consuming and may not produce a positive diagnosis in the presence of low rates of blood loss less than 0.5 to 1 ml/min. Nuclear imaging studies with radiolabelled red cells are useful to identify the site of haemorrhage. This test is also time consuming and is not applicable to patients who are haemodynamically unstable.

The discovery of malrotation at laparotomy was unexpected. Malrotation reportedly occurs in 1 in 500 live births, with over 80% presenting within the first month of life[5]. The true prevalence of malrotation in the adult population is unknown, although it is a finding on 1 in 500 gastrointestinal contrast studies[6]. The mesentery of the malrotated bowel is more tortuous, making the vascular supply more precarious. Patients typically present with signs of obstruction, intestinal ischaemia or haemorrhage[7]. An association between small intestinal malrotation and haemorrhage from a localised dilatation of the ileum has been reported previously in young adults and children under 10 years[8]. The pathogenesis of the haemorrhage from this dilated ileum is unknown. Functional obstruction within the aperistaltic segment of ileum may cause stasis of intestinal contents, leading to localised mucosal ulceration and subsequently haemorrhage[9].

The patient presented above had no evidence of localised bowel dilatation and no angiodysplasia was found on histology. He presented with life-threatening haemorrhage. Iron deficiency pointed towards prior undetected chronic intestinal blood loss. Laparotomy was undertaken due to cardiovascular instability. At laparotomy, we pursued a careful and systematic approach to isolate the bleeding segment of small bowel. By marking the upper limit of intra-luminal blood and using a series of small bowel clamps, we were able to confidently identify the site of haemorrhage. Further evaluation using intraoperative enteroscopy could have been undertaken if clinically indicated at the time. Reported success rates using this method are good, with detection of angiodysplasia in up to 46% of cases. However, endoscope-related trauma may create confusing findings and experience of its use in the emergency situation is very limited[3].

The precise pathophysiology of the bleeding in this case is uncertain. Histological examination showed dilated vessels within the jejunum wall, with erosions in the mucosal layer. This may have occurred due to localised hypertension, mechanically caused by the tortuosity of the blood vessels, kinking of the mesentery and venous congestion. There was no history of NSAID use and no frank ulceration was seen at histological examination. The patient had a low ferritin, suggesting that he may have suffered from episodes of chronic concealed haemorrhage. He also had a previous history of undiagnosed abdominal pain. CT scan had previously demonstrated diverticular disease. At retrospective review of these scans after laparotomy subtle evidence of malrotation was noted, with signs of swirling superior mesenteric vessels and abnormal rotation of the proximal jejunum distal to the duodeno-jejunal flexure. An association has been reported previously between congenital malrotation presenting in adult life and chronic abdominal pain[10].

The successful resolution of the patient's bleeding episode following operation encourages us to believe that release of the malrotated bowel and resection of the proximal jejunum was the correct course of treatment.

## Conclusion

We believe this report highlights an important aetiology in patients with obscure gastrointestinal haemorrhage. If a high index of suspicion is maintained, malrotation may be detected easily on axial imaging, such as CT scan, or small bowel contrast series. Careful intraoperative observation of small bowel haemorrhage followed by segmental resection is an effective method of treating life-threatening haemorrhage in unstable patients, in whom further investigation is not possible.

## Consent

Written informed consent was obtained from the patient for publication of this case report and any accompanying images. A copy of the written consent is available for review by the Editor-in-Chief of this journal.

## Competing interests

The authors declare that they have no competing interests.

## Authors' contributions

AB and DK performed the literature review and drafted the manuscript. PP provided the figures and helped to draft the manuscript. KMS conceived of the study, supervised the care of the patient, provided the clinical details, critically reviewed and helped to draft the manuscript. All authors read and approved the final manuscript.
